# Desmoplastic Spitz Nevus of the Thigh in a Young Woman: Clinical Presentation and Histopathological Diagnosis

**DOI:** 10.7759/cureus.103250

**Published:** 2026-02-09

**Authors:** Nicolas Opazo, Diego I Mendez-Villanueva, Camila Gacitúa, Manuel Rodríguez, Paulina Naranjo

**Affiliations:** 1 Dermatology, Hospital El Pino, Santiago, CHL; 2 Dermatology, Clínica Orlandi, Santiago, CHL; 3 Medicine and Surgery, Universidad de Santiago de Chile, Santiago, CHL

**Keywords:** dermatopathology, desmoplastic melanoma, desmoplastic nevus, spitz nevus, spitzoid tumor

## Abstract

Desmoplastic Spitz nevus is an uncommon variant within the spectrum of spitzoid melanocytic tumors and represents a well-known diagnostic challenge due to its histopathological resemblance to desmoplastic melanoma, a malignant entity associated with poor prognosis. We report the case of a 35-year-old woman who presented with a small, erythematous, firm nodule on the right thigh with progressive growth over four months. Complete surgical excision was performed. Histopathological examination revealed a dermal melanocytic proliferation composed of Spitz-type cells arranged in nests and sheets within a markedly desmoplastic stroma, with evidence of maturation in depth and absence of significant cytologic atypia or mitotic activity. Surgical margins were free of involvement, and the findings were consistent with a desmoplastic Spitz nevus. This case highlights the importance of recognizing the distinctive clinical and histopathological features of desmoplastic Spitz nevus to avoid misdiagnosis and unnecessary aggressive treatment. Accurate differentiation from desmoplastic melanoma is essential, and in ambiguous cases, immunohistochemical studies may be required to support the diagnosis.

## Introduction

Desmoplastic Spitz nevus is a rare variant within the spectrum of spitzoid melanocytic tumors. Clinically, it usually presents as a firm, symmetric, asymptomatic papule or nodule, most frequently located on the extremities of young adults, with a predilection for women in the third decade of life [1]. Although benign, its clinical relevance lies in its striking histopathological resemblance to desmoplastic melanoma, a malignant neoplasm associated with aggressive behavior and unfavorable prognosis [2].

This morphological overlap may lead to diagnostic uncertainty and potential overtreatment. Therefore, awareness of the characteristic clinical, histological, and immunohistochemical features of desmoplastic Spitz nevus is essential for accurate diagnosis and appropriate management. From a clinical standpoint, the differential diagnosis of a firm erythematous nodular lesion includes desmoplastic melanoma, dermatofibroma, intradermal or spindle cell nevus, adnexal tumors, and other fibrous or amelanotic cutaneous neoplasms. We report a case of desmoplastic Spitz nevus arising on the thigh of a young woman and discuss the main diagnostic criteria that allow differentiation from desmoplastic melanoma.

## Case presentation

**Figure 1:**
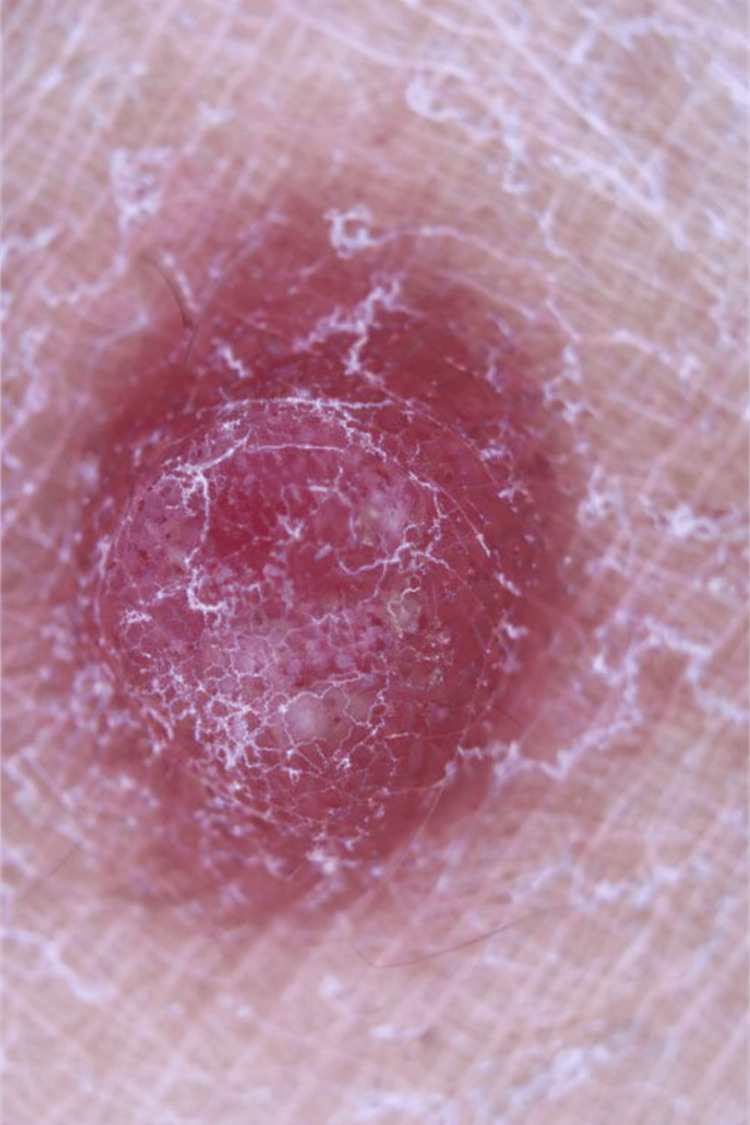
Dermoscopic image showing a small erythematous nodular lesion with superficial scaling.

A 35-year-old woman with no relevant medical history presented with a lesion on the right thigh that had been present for four months. The patient reported occasional pruritus and progressive growth of the lesion. Physical examination revealed a firm, erythematous, well-circumscribed nodule measuring approximately 4 mm in diameter, with a smooth surface and a central keratotic area, without ulceration (Figure 1).

Dermoscopic examination was nonspecific. Given the lesion's clinical characteristics and recent growth, complete surgical excision was performed for histopathological evaluation.

**Figure 2:**
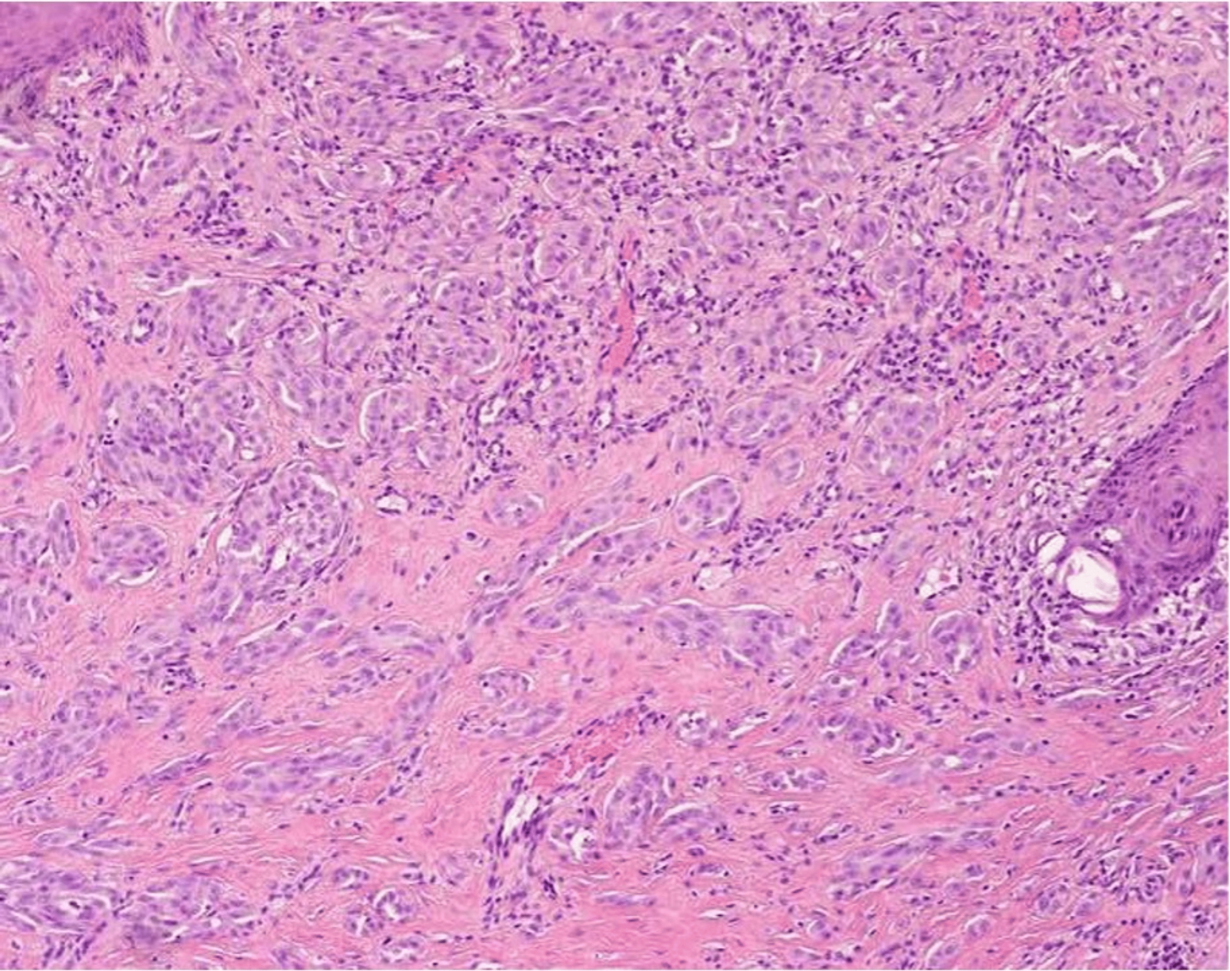
Histopathological section (10× magnification) demonstrating a dermal melanocytic proliferation composed of homogeneous Spitz-type cells with abundant eosinophilic cytoplasm and regular nuclei, arranged within a markedly desmoplastic stroma.

Microscopic examination demonstrated a dermal melanocytic proliferation composed of Spitz-type cells arranged in nests and sheets, showing an adnexocentric growth pattern within a markedly desmoplastic stroma. The epidermis exhibited compact hyperkeratosis. Progressive maturation of melanocytes with depth was observed, without significant cytologic atypia or mitotic figures. Surgical margins were free of involvement (Figure 2). Based on these findings, the diagnosis of desmoplastic Spitz nevus was established.

## Discussion

Desmoplastic Spitz nevus represents a distinctive histopathological variant of Spitz nevus characterized by a dense collagenous stroma and a predominantly or exclusively dermal growth pattern. Since its initial description, there has been debate as to whether this lesion represents a separate entity or a variant of classic Spitz nevus. Mackie and Doherty proposed desmoplastic Spitz nevus as a distinct histological entity due to its lack of junctional nests and its unique architectural features [3].

Clinically, desmoplastic Spitz nevus most commonly affects young women and tends to arise on the extremities, particularly the lower limbs [4]. The clinical presentation in our patient is consistent with previous reports describing small, firm, erythematous nodules in similar demographic groups. However, this nonspecific presentation overlaps with several other benign and malignant cutaneous tumors, making microscopic examination essential for accurate diagnosis.

Histopathological evaluation is crucial for diagnosis. Key features supporting desmoplastic Spitz nevus include lesion symmetry, well-circumscribed borders, progressive maturation of melanocytes with depth, dense collagenous stroma, and absence of significant cytologic atypia or mitotic activity [1,3]. In contrast, desmoplastic melanoma, which represents the most critical diagnostic consideration given its aggressive behavior, typically shows asymmetry, infiltrative growth, marked cytologic atypia, increased mitotic activity, lack of maturation, and poorly defined margins. 

Immunohistochemical studies can be helpful in challenging cases. Desmoplastic Spitz nevi usually demonstrate diffuse positivity for S-100 protein, decreasing expression of Human Melanoma Black-45 (HMB-45) toward the deeper portions of the lesion, and a low proliferative index assessed by Ki-67, typically below 2% [4,5]. Conversely, desmoplastic melanoma often exhibits a higher proliferative index and is frequently negative for melanocytic differentiation markers such as HMB-45 and Melan-A [4,6].

Additional differential diagnoses include sclerosing blue nevus and dermatofibroma, which can generally be distinguished based on morphology and immunohistochemical profile [2,5]. Sclerosing blue nevus is characterized by the presence of heavily pigmented dendritic melanocytes embedded within a fibrotic stroma and lacks the epithelioid or spindle cell cytology typical of Spitz lesions [2]. Dermatofibroma, in contrast, shows a fibroblastic proliferation, epidermal hyperplasia, and absence of melanocytic differentiation [5]. Neurofibroma may also be considered due to its spindle cell morphology; however, it typically exhibits a diffuse growth pattern composed of wavy spindle cells and lacks melanocytic features [2,5].

In the present case, the combination of clinical context and characteristic histopathological features was sufficient to establish the diagnosis without the need for ancillary immunohistochemical studies. Accurate recognition of desmoplastic Spitz nevus is essential to prevent misdiagnosis and avoid unnecessary aggressive surgical or oncologic management.

## Conclusions

Desmoplastic Spitz nevus is a benign melanocytic lesion that may closely mimic desmoplastic melanoma both clinically and histologically, posing a relevant diagnostic challenge. Careful assessment of clinical presentation combined with detailed histopathological evaluation is essential to achieve an accurate diagnosis. Awareness of this uncommon variant allows clinicians and pathologists to distinguish it from malignant counterparts, thereby preventing overtreatment and unnecessary patient morbidity. Early recognition and correct classification play a key role in ensuring appropriate management and favorable outcomes.
